# Identification of core genes and clinical roles in pregnancy-associated breast cancer based on integrated analysis of different microarray profile datasets

**DOI:** 10.1042/BSR20190019

**Published:** 2019-06-25

**Authors:** Jiao Zhang, Yan-Jun Zhou, Zhi-Hao Yu, Ao-Xiang Chen, Yue Yu, Xin Wang, Xu-Chen Cao

**Affiliations:** 1 The First Department of Breast Cancer, Tianjin Medical University Cancer Institute and Hospital, National Clinical Research Center for Cancer, Tianjin 300060, China; 2 Key Laboratory of Cancer Prevention and Therapy, Tianjin 300060, China; 3 Tianjin’s Clinical Research Center for Cancer, Tianjin 300060, China; 4 Key Laboratory of Breast Cancer Prevention and Therapy, Tianjin Medical University, Ministry of Education, Tianjin 300060, China

**Keywords:** bioinformatical analysis, differentially expressed genes, pregnancy-associated breast cancer, prognosis

## Abstract

More women are delaying child-birth. Thus, the diagnosis of pregnancy-associated breast cancer (PABC) will continue to increase. The aim of this study was to identify core candidate genes of PABC, and the relevance of the genes on the prognosis of PABC. GSE31192 and GSE53031 microarray profile datasets were downloaded from the Gene Expression Omnibus database and differentially expressed genes were analyzed using the R package and GEO2R tool. Then, Gene Ontology and Kyoto Encyclopedia of Gene and Genome pathway enrichment analyses were performed using the Database for Annotation, Visualization, and Integrated Discovery. Moreover, the Search Tool for the Retrieval of Interacting Genes and the Molecular Complex Detection Cytoscape software plug-in were utilized to visualize protein–protein interactions and to screen candidate genes. A total of 239 DEGs were identified in PABC, including 101 up-regulated genes mainly enriched in fatty acid activation and the fibroblast growth factor signaling pathway, while 138 down-regulated genes particularly involved in activation of DNA fragmentation factor and apoptosis-induced DNA fragmentation. Fourteen hub genes with a high degree of connectivity were selected, including CREB1, ARF3, UBA5, SIAH1, KLHL3, HECTD1, MMP9, TRIM69, MEX3C, ASB6, UBE2Q2, FBXO22, EIF4A3, and PXN. Overall survival (OS) analysis of core candidate genes was performed using the Gene Expression Profiling Interactive Analysis and UALCAN websites. High ASB6 expression was associated with worse OS of PABC patients. Molecular subtypes and menopause status were also associated with worse OS for PABC patients. In conclusion, ASB6 could be a potential predictor and therapeutic target in patient with PABC.

## Introduction

Pregnancy-associated breast cancer (PABC) refers to breast cancer that is diagnosed during gestation or within 1 year of delivery [1–3]. Pregnancy-related disease occurs in 0.2–3.8% of all breast cancer patients, comprising 1:10000 to 1:3000 pregnancies. The prevalence is much higher in pre-menopausal women, with 10–25% of breast cancers estimated to be pregnancy-associated [4–9].

Although breast cancer is the most frequently diagnosed malignancy among women and is increasing in incidence, PABC has remained a rare event in the last decade [5,8,10,11]. However, PABC has increased more recently as the government policy in China allows the birth of a second child and given the trend of women in postponing pregnancy [12–15]. While PABC formation and progression has been well-studied, the biology of PABC is still not clear. The poor prognosis of PABC has been attributed to a higher risk for nodal involvement, higher hormonal milieu, and more frequent delay in diagnosis [3,16–20]. However, several studies found no major differences in the expression of hormone receptors and a more common association with the high expression of potentially relevant cancer targets, such as insulin growth factor, Wnt/β-catenin and RANK ligand, and low prevalence of tumor-infiltrating lymphocytes [21–25]. There have been considerable efforts in managing breast cancer that develops during pregnancy. Yet, very little progress has been in understanding of the biology of this disease. This challenging scenario in PABC demands knowledge of the causes and potential molecular mechanisms of PABC, and molecular biomarkers for the early diagnosis and individualized therapy.

Gene microarray profiling is widely used in life sciences. This approach can quickly detect all the expressed genes within the same tissue and reveals differentially expressed genes (DEGs) [26]. The use of the gene chip can uncover thousands of DEGs associated with different biological processes, different signal pathways, or molecular functions of cancer. The popularity of gene microarray profiling has generated a huge amount of data that is available in public databases. These data can be scrutinized to integrate and re-analyze gene expression profiles, provide new methods, and obtain valuable clues for the disease of interest.

There are many reports that integrate gene expression profiles on breast cancer, however, hard to see it in PABC [27–29]. In this study, we screened two microarray profile datasets, GSE31192 [30] and GSE53031 [31], from the National Center for Biotechnology Information Gene Expression Omnibus database (NCBI-GEO). Then, we filtered DEGs using combined GEO2R and R package analyses, and performed Gene Ontology (GO) and Kyoto Encyclopedia of Gene and Genome (KEGG) pathway enrichment analyses of DEGs with the Database for Annotation, Visualization, and Integrated Discovery (DAVID). The protein–protein interaction (PPI) network of DEGs developed with the Search Tool for the Retrieval of Interacting Genes (STRING) and top modular analysis with Cytoscape software were used to identify core candidate genes in PABC. The association of the core candidate genes with overall survival (OS) was assessed using the Gene Expression Profiling Interactive Analysis (GEPIA) and UALCAN websites. The bioinformatics analyses revealed a number of DEGs. In particular, ASB6 (ankyrin repeat and suppressor of cytokine signaling (SOCS) box containing 6) could be potentially considered as a biomarker in PABC.

## Materials and methods

### Acquisition microarray datasets

Gene expression microarray data were obtained from the GSE31192 and GSE53031 microarray profile datasets in the NCBI-GEO database (http://www.ncbi.nlm.nih.gov/geo/), which is a public and freely available platform. GSE31192 is based on the GPL570 platform of the Affymetrix Human Genome U133 Plus 2.0 Array, which includes 20 PABC samples containing six cancer epithelial tissues, six cancer stroma tissues, three normal epithelial tissues, and five normal stroma tissues, and 13 non-PABC control samples that contained four cancer epithelial tissues, four cancer stroma tissues, three normal epithelial tissues, and two normal stroma tissues. GSE53031 is based on the GPL13667 platform of the Affymetrix Human Genome U219 Array, and contains 54 PABC tissues and 113 non-PABC control tissues.

### Detection processing of DEGs

GEO2R (Online: https://www.ncbi.nlm.nih.gov/geo/geo2r/) is an online tool that can compare two or more groups of samples in the GEO series datasets. A *P*-value < 0.05 and |log FC| > 1 were set as the cut-off criteria. For the integrated analysis of the high-throughput functional genomic expression data, the raw data were processed in R according to the |log FC| > 1 and *q*-value < 0.05 criteria. The combination of GEO2R and R was used to select DEGs. The biological coefficient of variation, which was the square-root of the dispersions, was set to 0.01 following the suggestion in the R manual and Volcano plots was constructed [32]. Datasets that separated up-regulated and down-regulated DEGs were uploaded into FunRich version 3.1.3, a free software for functional enrichment analysis of gene sets that contains additional features [33]. Venn diagram of the analysis results plotted commonly changed DEGs using the *P* < 0.05 and |log FC| > 1 criteria.

### GO and KEGG pathway analysis of DEGs

GO and KEGG pathway enrichment analyses were performed to detect the biological functions and potential pathway of DEGs. GO is a commonly used method to annotate genes and gene products, and identifies characteristic biological attributes for high-throughput genome or transcriptome data [34]. KEGG is a collection of databases dealing with genomes, biological pathways, diseases, drugs, and chemical substances [35,36]. GO and KEGG analyses were applied through the DAVID (Online: https://david.ncifcrf.gov/), a website bioinformatics resource that can provide tools for the gene annotation, visualization, and integrated discovery function [37]. The cut-off criterion was *P* < 0.05.

### PPI network and module analyses

Search Tool for the Retrieval of Interacting Genes (STRING, https://string-db.org) is an online tool designed to evaluate PPIs [38]. The DEGs involved in the PPI network were identified using STRING, with confidence network edges and a medium confidence of 0.400 as the product criteria. The Tab Separated Values format document was downloaded as a simple tabular text output. Cytoscape_v3.6.0 was used to construct the PPI network and analyze the interactions of the candidate DEGs encoding proteins [39]. The Molecular Complex Detection (MCODE) software application was used to screen the top modules in the PPI network with a degree cut-off = 2, node score cut-off = 0.2, k-core = 2, and maximum depth = 100. The corresponding proteins in the central nodes and highly degree were potential core proteins encoded by key candidate genes that have important physiological regulatory functions.

### OS analysis of core candidate genes in PABC

GEPIA (http://gepia.cancer-pku.cn/) is a web-based tool that rapidly delivers customizable functionalities based on TCGA and GTEx data. Patient survival analysis can be conducted [40]. UALCAN (Online: http://ualcan.path.uab.edu/index.html) is a user-friendly, interactive web resource used to analyze cancer transcriptome data. UALCAN allows users to identify up- and down-regulated expressed genes in individual cancer types, as well as to estimate the effect of gene expression level and clinicopathologic features on patient survival [41]. Presently, GEPIA was used to determine OS related to core candidate genes that had been identified. The effects of the expression of the core candidate genes on PABC OS according to molecular subtypes and menopause status were assessed by UALCAN and *P*-values were calculated.

## Results

### Identification of DEGs in PABC

**Figure 1:**
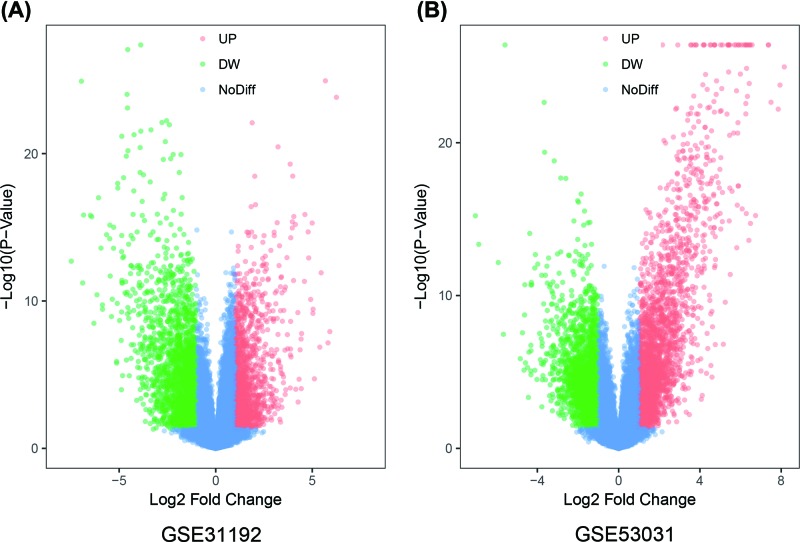
Analysis of the differentially expression genes between the PABC and non-PABC using Volcano plots Red and green points represent up-regulated and down-regulated genes. No differentially expressed genes in blue points. (**A**) DEGs based on GSE31192. (**B**) DEGs based on GSE53031.

**Figure 2:**
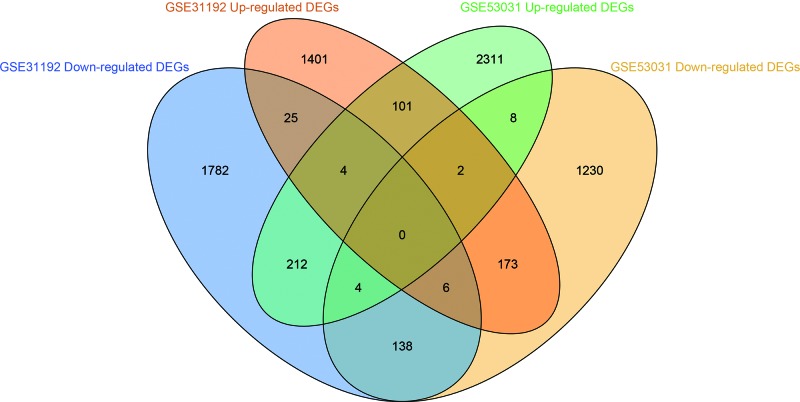
Identification of 239 (101 up-regulated and 138 down-regulated) commonly changes differentially expression genes from GSE31192 and GSE53031 microarray profile datasets Different color areas stand for up-regulated and down-regulated genes, which were defined with *P* < 0.05 and |log FC| > 1 as the cut-off criterion.

NCBI-GEO is a free database of microarray profiles and next-generation sequencing data. The database was used to obtain PABC, non-PABC, and normal or adjacent stroma tissue gene expression profiles of the GSE31192 and GSE53031 datasets. A total of 1712 and 2642 DEGs were respectively up-regulated in these two datasets, while 2171 and 1388 DEGs were respectively down-regulated using *P*<0.05 and |logFC|>1 as the cut-off criteria (Figures 1 and 2). After integrated bioinformatic analysis, 239 common DEGs were identified from the two profile datasets. These included 101 up-regulated genes and 138 down-regulated genes in PABC, compared with non-PABC (Figure 2 and Table 1).

**Table 1:** 239 differentially expressed genes were identified from two profile datasets, including 101 up-regulated genes and 138 down-regulated genes in PABC

DEGs	Genes name
Up-regulated	ASB6, GRIN2C, NOL8, TMEM97, TMEM132E, C11orf16, PDE11A, OR10A4, DIP2A, C15orf62, GRK5, RGS12, KRI1, SLC2A6, CD209, GGA1, CXCR5, VARS, PARVB, STAT5B, TBKBP1, UBASH3B, SOX6, RIN1, ATP6AP1L, BEST1, FOXRED2, ACSBG1, FCRLA, CPEB1, PLCG1, POLR2J4, GTDC1, MGAT4B, LSS, GRAPL, DISP1, MUC20, POU2F2, KCNJ16, BTN3A1, KIR2DL5A, PXN, PDE10A, LRCH3, DBF4B, AK2, PLEKHO2, PTPN7, PGBD5, ZDHHC3, FCRL2, TRIM69, RPS28, FMNL1, ADCY7, HLA-DOB, KCNIP2, CRYGS, LRRC28, GNL1, TOMM40L, AIF1, CCDC146, AP4B1, FGD2, FAM129C, SLCO3A1, DMRT3, IRF1, RAB7B, C8orf34, ADAMTSL1, LOC81691, SLC19A3, TNFRSF9, KLHL3, ACRC, ZNF93, AGFG2, CIDEC, TAP2, IL12RB1, SEZ6L, SGK494, CD22, FAM124A, VWCE, TBC1D1, THOC5, ITGB7, KCNJ10, APOBEC3G, SRGAP2, APOBEC3B, ACSL6, CENPN, CD274, TNFSF11, MMP9, CXCL13
Down-regulated	FSIP1, RAB30, AR, RERG, COPB1, ACADSB, TPD52, DGUOK, ZNF33A, SYTL2, EPB41L5, WBP11, GLRB, ZNF33B, TRERF1, SLC25A15, AIMP1, AIM1, COX11, APLP2, RPRD1A, FBXO22, SLC19A2, CUL4A, SFXN2, FAM83H, ANO1, ZNF8, ZBTB38, ANG, ABHD12, MORF4L2, MICALCL, KDM4B, ZNF639, CCT8, ZNF562, ARF3, TBC1D12, SRPK2, ABI2, RAB14, BMPR1A, GFPT1, NDFIP2, G2E3, TIA1, DPY19L4, SCOC, NCOR1, TRUB1, SMARCA1, IFNAR1, PLEKHA1, ZADH2, USP14, TP53I11, KLHL8, VPS54, NUDT19, APOOL, ZNF551, PEX13, RPF1, AKT1S1, TSPYL1, DOCK1, TMEM106B, SS18, WDR61, YIPF6, REL, RAB11A, GNAQ, MIB1, SLC12A6, NOM1, SPCS2, ARMCX1, RMND5A, SEC23B, RNF103, TMEM33, EPS15L1, UBA5, PPP4R1, AKTIP, RALBP1, FBXO11, CEP290, ZNF664, FAR1, GTF2A2, ZRANB1, EIF4A3, RUFY2, ZNF302, RALGAPA1, HMGB1, KLF3, CTBS, ZNF3, EXOSC3, AKT2, GLUD1, MEX3C, UBE2Q2, KDELR2, CLIP1, SCARB2, SMARCE1, HIBADH, EIF1AX, STRN3, IER3IP1, HINT1, GFM1, SLK, PAN3, ZNF24, ZMYM2, SECISBP2, CREB1, PRPF39, NEK4, CDKN2B, NPTN, TWF1, KPNA1, OSBPL9, ZYG11B, SNRPB2, CCDC90B, HECTD1, MGST2, SIAH1, DNM1L, PICALM

### DEGs GO and KEGG pathway enrichment analyses

**Figure 3:**
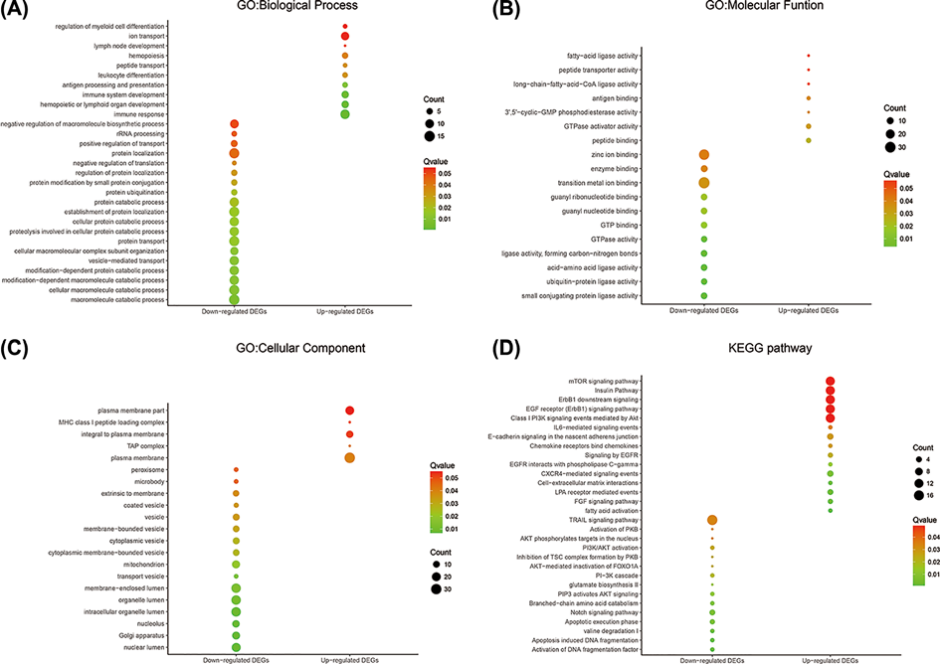
Biology function and signaling pathway analysis of DEGs in PABC (**A**) GO significant enriched of biological process. (**B**) GO significant enriched of molecular function. (**C**) GO significant enriched of Cellular Component. (**D**) KEGG significant enriched of pathway.

For a more in-depth understanding of the screened DEGs, we analyzed the GO and KEGG pathway enrichment of the identified DEGs through DAVID. All the DEGs were imported to DAVID and GO analysis comprised three functional groups: biological process (BP) group, molecular function (MF) group, and cellular component (CC) group. As shown in Figure 3A–C and Table 2, the GO BP group analysis indicated that the up-regulated genes were mainly enriched in the immune response, hemopoietic or lymphoid organ development, immune system development, antigen processing, and presentation and leukocyte differentiation. The down-regulated genes were mainly enriched in the macromolecule catabolic process, cellular macromolecule catabolic process, modification-dependent protein catabolic process, modification-dependent macromolecule catabolic process, and vesicle-mediated transport (Figure 3A). For the GO MF group, the up-regulated genes were mainly enriched in peptide binding, GTPase activator activity, 3′,5′-cyclic-GMP phosphodiesterase activity, antigen binding, and long-chain-fatty-acid-CoA ligase activity. The down-regulated genes were mainly enriched in small conjugating protein ligase activity, ubiquitin protein ligase activity, acid-amino acid ligase activity, ligase activity and formation of carbon-nitrogen bonds, and GTPase activity (Figure 3B). Concerning the GO CC group, the up-regulated genes were mainly enriched in plasma membrane, TAP complex, MHC class I peptide loading complex, plasma membrane, and integral to plasma membrane. The down-regulated genes were mainly enriched in nuclear lumen, Golgi apparatus, nucleolus, intracellular organelle lumen, and organelle lumen (Figure 3C).

**Table 2:** Genes ontology enrichment analysis of differentially expressed genes in PABC

DEGs	Category	Term	Count	Fold enrichment	*P*-value	FDR
Up-regulated	GOTERM_BP_DIRECT	GO:0006955∼immune response	12	3.17	0.00	1.66
	GOTERM_BP_DIRECT	GO:0048534∼hemopoietic or lymphoid organ development	7	4.92	0.00	4.10
	GOTERM_BP_DIRECT	GO:0002520∼immune system development	7	4.63	0.00	5.46
	GOTERM_BP_DIRECT	GO:0019882∼antigen processing and presentation	4	8.81	0.01	7.57
	GOTERM_BP_DIRECT	GO:0002521∼leukocyte differentiation	4	5.58	0.03	14.34
	GOTERM_MF_DIRECT	GO:0042277∼peptide binding	5	4.38	0.02	8.76
	GOTERM_MF_DIRECT	GO:0005096∼GTPase activator activity	5	4.04	0.03	5.52
	GOTERM_MF_DIRECT	GO:0047555∼3′,5′-cyclic-GMP phosphodiesterase activity	12	50.81	0.03	9.21
	GOTERM_MF_DIRECT	GO:0003823∼antigen binding	3	9.52	0.03	9.49
	GOTERM_MF_DIRECT	GO:0004467∼long-chain-fatty-acid-CoA ligase activity	2	35.56	0.04	10.89
	GOTERM_CC_DIRECT	GO:0005886∼plasma membrane	31	1.36	0.03	8.79
	GOTERM_CC_DIRECT	GO:0042825∼TAP complex	2	47.42	0.03	9.15
	GOTERM_CC_DIRECT	GO:0042824∼MHC class I peptide loading complex	2	36.88	0.04	7.21
	GOTERM_CC_DIRECT	GO:0044459∼plasma membrane part	20	1.50	0.04	8.84
	GOTERM_CC_DIRECT	GO:0005887∼integral to plasma membrane	12	1.67	0.04	8.27
Down-regulated	GOTERM_BP_DIRECT	GO:0009057∼macromolecule catabolic process	15	2.18	0.00	12.00
	GOTERM_BP_DIRECT	GO:0044265∼cellular macromolecule catabolic process	14	2.19	0.01	15.46
	GOTERM_BP_DIRECT	GO:0019941∼modification-dependent protein catabolic process	12	2.37	0.01	16.88
	GOTERM_BP_DIRECT	GO:0043632∼modification-dependent macromolecule catabolic process	12	2.37	0.01	16.88
	GOTERM_BP_DIRECT	GO:0016192∼vesicle-mediated transport	12	2.36	0.01	17.26
	GOTERM_MF_DIRECT	GO:0019787∼small conjugating protein ligase activity	9	6.12	1.05E-04	0.14
	GOTERM_MF_DIRECT	GO:0004842∼ubiquitin-protein ligase activity	8	6.14	3.10E-04	0.41
	GOTERM_MF_DIRECT	GO:0016881∼acid-amino acid ligase activity	9	5.05	3.89E-04	0.52
	GOTERM_MF_DIRECT	GO:0016879∼ligase activity, forming carbon-nitrogen bonds	9	4.39	9.70E-04	1.29
	GOTERM_MF_DIRECT	GO:0003924∼GTPase activity	8	4.28	0.00	3.40
	GOTERM_CC_DIRECT	GO:0031981∼nuclear lumen	21	1.85	0.00	8.61
	GOTERM_CC_DIRECT	GO:0005794∼Golgi apparatus	15	2.19	0.00	8.63
	GOTERM_CC_DIRECT	GO:0005730∼nucleolus	13	2.38	0.00	9.14
	GOTERM_CC_DIRECT	GO:0070013∼intracellular organelle lumen	24	1.72	0.00	9.95
	GOTERM_CC_DIRECT	GO:0043233∼organelle lumen	24	1.68	0.01	12.81

Abbreviations: BP, biological process; CC, cellular component; Count, the number of enriched genes in each term; FDR, false discovery rate; MF, molecular function.

In addition, functional and signaling pathway enrichment of the DEGs was conducted. The up-regulated genes were mainly enriched in fatty acid activation, fibroblast growth factor signaling pathway, lysophosphatidic acid receptor mediated events, cell-extracellular matrix interactions, and CXCR4-mediated signaling events. The down-regulated genes were mainly enriched in activation of DNA fragmentation factor, apoptosis-induced DNA fragmentation, valine degradation I, apoptotic execution phase, and Notch signaling pathway (Figure 3D and Table 3).

**Table 3:** Signaling pathway enrichment analysis of differentially expressed genes in PABC

DEGs	Biological pathway	Count	*P*-value	Mapped gene names
Up-regulated	Fatty acid activation	2	0.00	ACSBG1, SL6
	FGF signaling pathway	3	0.00	STAT5B, CG1, MP9
	LPA receptor mediated events	4	0.00	PLCG1, XN, DCY7, MP9
	Cell-extracellular matrix interactions	2	0.00	PARVB, XN
	CXCR4-mediated signaling events	5	0.00	STAT5B, LCG1, XN, TPN7, MP9
	EGFR interacts with phospholipase C-gamma	2	0.01	PLCG1, DCY7
	Signaling by EGFR	3	0.02	PLCG1, XN, DCY7
	Chemokine receptors bind chemokines	2	0.02	CXCR5, XCL13
	E-cadherin signaling in the nascent adherens junction	5	0.02	STAT5B, LCG1, DCY7, TGB7, MMP9
	IL6-mediated signaling events	2	0.03	IRF1, TNFSF11
Down-regulated	Activation of DNA fragmentation factor	2	0.00	HMGB1, KPNA1
	Apoptosis induced DNA fragmentation	2	0.00	HMGB1, KPNA1
	Valine degradation I	2	0.00	ACADSB, HIBADH
	Apoptotic execution phase	3	0.00	HMGB1, KPNA1, DNM1L
	Notch signaling pathway	4	0.00	AR, NCOR1, RAB11A, MIB1
	Branched-chain amino acid catabolism	2	0.00	ACADSB, HIBADH
	PIP3 activates AKT signaling	2	0.01	AKT1S1, CREB1
	Glutamate biosynthesis II	1	0.01	GLUD1
	PI-3K cascade	2	0.02	AKT1S1, CREB1
	AKT-mediated inactivation of FOXO1A	1	0.02	CREB1

Count: the number of enriched genes in each term.

### DEGs PPI network and modular analysis

**Figure 4:**
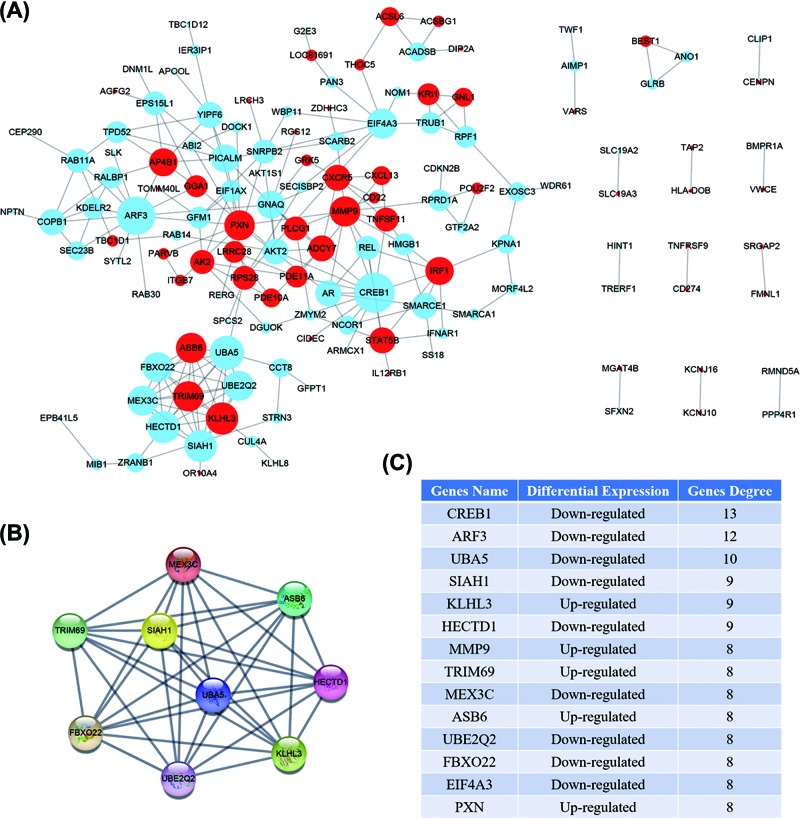
DEGs PPI network complex and the module analysis Up-regulated genes were marked in red, down-regulated genes were marked in light blue, and the lines show the interaction between the DEGs. A node degree is defined as the number of other nodes connected to the node. (**A**) DEGs were filtered into PPI network complex using STRING online database and Cytoscape software. (**B**) The module which the most highlighted circle areas. (**C**) High degree nodes value and genes name.

Using the STRING online database, a total of 139 DEGs (54 up-regulated and 85 down-regulated genes) of the 239 commonly altered DEGs were filtered into the PPI network complex. One hundred of the 239 DEGs did not fall into the PPI network. Based on the STRING output and Cytoscape software analysis, 54 nodes and 131 edges were identified in up-regulated DEGs and 85 nodes and 188 edges in down-regulated DEGs. The PPI network complex defaulter filter degree was between 1 and 20 inclusive (Figure 4A).

According to the degree of genes and the module was selected using the MCODE Cytoscape plug-in, the most significant fourteen node degree genes identified using a filtering degree >7 as the criterion were CREB1, ARF3, UBA5, SIAH1, KLHL3, HECTD1, MMP9, TRIM69, MEX3C, ASB6, UBE2Q2, FBXO22, EIF4A3, and PXN (Figure 4B,C). The proteins of these central nodes might be the core proteins and the genes could be key candidate genes with important roles in the progress of PABC and prognosis. They could be potential therapeutic targets.

### Core candidate genes OS analysis

**Figure 5:**
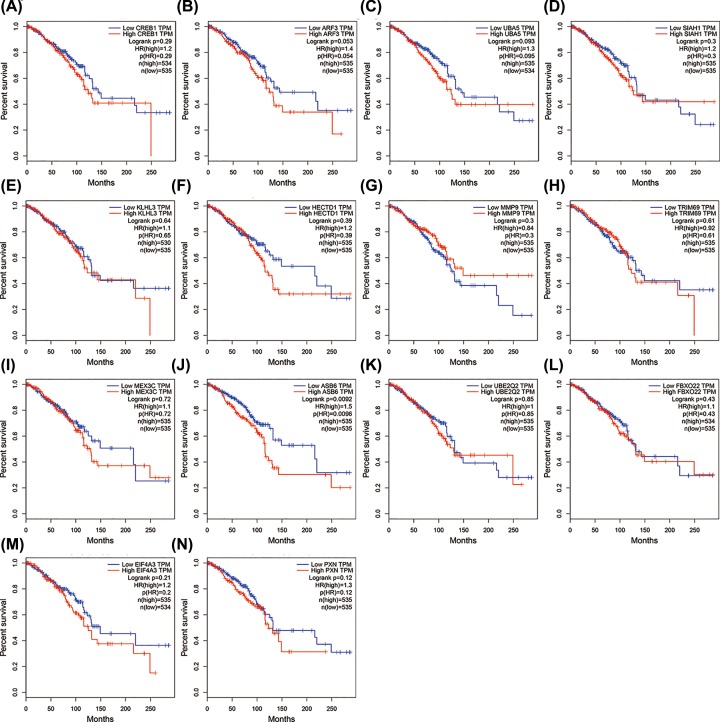
Overall survival values of core candidate genes in pregnancy-associated breast cancer (**A**) CREB1 (204314_s_at; *P* = 0.29). (**B**) ARF3 (200734_s_at; *P* = 0.053). (**C**) UBA5 (222579_at; *P* = 0.093). (**D**) SIAH1 (202981_x_at; *P* = 0.3). (**E**) KLHL3 (221221_s_at; *P* = 0.64). (**F**) HECTD1 (241683_at; *P* = 0.39). (**G**) MMP9 (203936_s_at; *P* = 0.3). (**H**) TRIM69 (229037_at; *P* = 0.61). (**I**) MEX3C (1556873_at; *P* = 0.72). (**J**) ASB6 (221657_s_at; *P* < 0.01). (**K**) UBE2Q2 (224747_at; *P* = 0.85). (**L**) FBXO22 (225736_at; *P* = 0.43). (**M**) EIF4A3 (201303_at; *P* = 0.21). (**N**) PXN (201087_at; *P* = 0.12).

The prognostic information of the 14 core candidate genes is freely available in http://gepia.cancer-pku.cn. High expression of ASB6 (*P* < 0.01) was associated with worse OS of PABC patients. No difference on OS was evident with the high expression of KLHL3 (*P* = 0.64), MMP9 (*P* = 0.3), TRIM69 (*P* = 0.61), and PXN (*P* = 0.12), or with the low expression of CREB1 (*P* = 0.29), ARF3 (*P* = 0.053), UBA5 (*P* = 0.093), SIAH1 (*P* = 0.3), HECTD1 (*P* = 0.39), MEX3C (*P* = 0.72), UBE2Q2 (*P* = 0.85), FBXO22 (*P* = 0.43), and EIF4A3 (*P* = 0.21) (Figure 5A–N).

**Figure 6:**
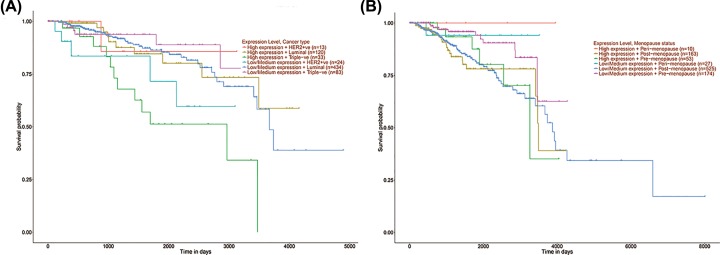
Effect of core candidate genes expression level in molecular subtypes and menopause status on pregnancy-associated breast cancer overall survival (**A**) ASB6 expression level effect OS of different subtypes on PABC (*P*  < 0.01). (**B**) ASB6 expression level effect OS of different menopause status on PABC (*P* = 0.02).

Of the 14 identified genes, the high expression of ASB6 was associated with worse OS for PABC patients according to molecular subtypes and menopause status. Compared with low expression of ASB6 in triple negative PABC, high expression of ASB6 was associated with worse OS for PABC patients, while the opposite result was obtained in the Her-2 overexpression subtype (Figure 6A). High expression of ASB6 was associated with poor OS in pre-menopausal PABC patients. By contrast, low expression of ASB6 was associated with a poor prognosis in peri-menopausal patients (Figure 6B). No statistical significance of ASB6 expression levels was evident according to luminal subtype and post-menopause status.

## Discussion

The prevalence of PABC is increasing, and the situation will become serious as the policy of planned birth has changed in China. Early diagnosis, prevention, and personalized therapy of PABC are vital and require more knowledge of the disease.

This study integrated two microarray profile datasets and utilized bioinformatics methods to identify 239 common DEGs. The genes comprised 101 up-regulated and 138 down-regulated genes. The 239 DEGs were divided into biological process, molecular function, and cellular component groups by GO and further clustered based on KEGG pathways with significant enrichment analysis. The up-regulated DEGs were mainly enriched in the immune response, peptide binding, plasma membrane, and fatty acid activation. The influences of fatty acids on cancer have been extensively studied, with variable influences apparent in different cancers [42–46]. The down-regulated DEGs were mainly enriched in the macromolecule catabolic process, small conjugating protein ligase activity, nuclear lumen, and activation of DNA fragmentation factor. Interestingly, the signaling pathway enrichment analysis indicated that epidermal growth factor receptor and protein kinase B, which is closely related to cell growth, proliferation, and apoptosis, play a critical role in cancer [47–52]. By reviewing the previous studies, Harvell et al. and Ma et al. found that PABC stroma tissue differentially expressed immune response genes, involved in angiogenesis and extracellular matrix deposition, associated with high-grade tumors [30,53]. Azim et al. and Muller et al. observed that CXCR4, belong to G protein-coupled receptor family of cell surface molecules, increase the metastatic potential of breast cancer cells during pregnancy [31,54]. These are consisted with our GO and KEGG enrichment results, will help us to further understand this cohort of patients.

Among the presently identified CREB1, ARF3, UBA5, SIAH1, KLHL3, HECTD1, MMP9, TRIM69, MEX3C, ASB6, UBE2Q2, FBXO22, EIF4A3, and PXN were identified as core genes that displayed a high degree of connectivity in the PPI network. These genes were expressed at higher or lower levels in PABC tissues. The up-regulation of ASB6 was significantly associated with worse OS of PABC patients. The same result was detected in triple negative molecular subtypes and pre-menopausal patients. Further analyses implicated ASB6 as having a central role in the biological process of PABC and in the prognosis. ASB6 might be a potentially valuable therapeutic molecular target.

ASB6 belongs to a family of ankyrin repeat proteins that contain a C-terminal SOCS box motif. ASB6 can couple to an adaptor protein containing PH and SH2 domains that interacts with insulin receptor to execute glucose transport [55,56]. Accumulating evidence suggests that the SOCS box acts as a bridge between specific substrate-binding domains and more generic proteins that comprise a large family of E3 ubiquitin protein ligases, which mediated the ubiquitination and subsequent proteasomal degradation of target proteins [57]. ASB6 up-regulation by Areca nut extracts has been identified in normal keratinocytes and oral cancer cells, and is highly associated with a worse prognosis of oral squamous cell carcinoma [58]. The authors also reported differential expression of ASB6 and its influence on prognosis, which are consistent with our findings. Whether these observations with ASB6 in two different types of cancer extend to other cancers remains to be determined.

In conclusion, this study identified DEGs through integrated bioinformatical analysis to find potential core candidate genes involved in PABC. ASB6 was identified as potentially having a pivotal role in the occurrence, development, and prognosis of PABC. ASB6 is thus implicated as a novel biomarker and potential therapeutic target.

## Abbreviations

ASB6: ankyrin repeat and suppressor of cytokine signaling box containing 6
DAVID: Database for Annotation, Visualization, and Integrated Discovery
DEG: differentially expressed gene
GEPIA: gene expression profiling interactive analysis
GO: gene ontology
KEGG: Kyoto Encyclopedia of Gene and Genome
MCODE: molecular complex detection
OS: overall survival
PABC: pregnancy-associated breast cancer
PPI: protein–protein interaction
SOCS: suppressor of cytokine signaling
STRING: Search Tool for the Retrieval of Interacting Genes
